# An Emerging Market: The Impact of User Selection on the Decision-Making Behavior of Mobile Medical Businesses in China

**DOI:** 10.3389/fpsyg.2022.723340

**Published:** 2022-02-21

**Authors:** Xinglong Xu, Jiajia Wei, Lulin Zhou, Henry Asante Antwi

**Affiliations:** ^1^School of Management, Jiangsu University, Zhenjiang, China; ^2^Medical Insurance and Public Policy Research Center, Jiangsu University, Zhenjiang, China

**Keywords:** mobile medical, decision-making behavior, user choice by substituting the probabilities, emerging market, China

## Abstract

**Background:**

User selection is an important guarantee for the sustainable development of mobile medical businesses. Under the background of increasingly fierce competition, the decision-making behavior of mobile medical businesses will directly affect the choice of the behavior of users.

**Methods:**

The study constructs the decision-making behavior model of mobile medical business based on the user choice and adds the role of people in government. It uses the game method to explore the relationship between the government, mobile medical business, and users. Finally, it makes an example analysis. Empirical research is conducted to demonstrate the influence of different parameter changes on the results.

**Results:**

The results show that in the absence of government intervention, users’ choice of filtering information will create a bad environment for mobile medical businesses, and further will be reduced, and the expected utility of businesses will not be affected causing a reduction in expected utility of companies. Similarly, government regulations can effectively improve the mobile medical environment and enhance the expected utility of mobile medical companies.

**Conclusion:**

The government needs to formulate relevant laws and regulations to ensure the orderly operation of the mobile medical market and strengthen government supervision. It is necessary to conduct publicity and education to protect the fundamental interests of users and businesses.

## Introduction

The Chinese government is committed to building mobile healthcare as an effective tool for integrating healthcare services and medical and health institutions at all levels. This includes mobile Internet, cloud computing, smart medical services, Internet, and other technologies to promote the level of health information to achieve the healthy China agenda. In 2020, China’s mobile medical market had more than 3.49 million apps, the user scale had reached 590 million people, and the market scale was close to 53.96 billion Yuan. As an emerging industry, the development prospect of mobile medical businesses is widely concerned and favored by the government and the general public. Presently, China’s mobile medical market is still market-oriented, and user demand is the fundamental guarantee for the operation and development of mobile medical businesses. However, businesses pay more attention to their economic profits in the decision-making process of application (APP) software development, promotion, and application. The nature of public welfare in the medical industry is diluted. Problems such as the excessive provision of medical services, unsafe information, unclear responsibilities, and rights of medical disputes often occur. Therefore, in the increasingly competitive mobile medical market, some of the important questions often asked are (1) What kind of decision-making behavior should mobile medical businesses take to create a good business environment to attract more users? (2) Under the different decision-making behaviors of businesses, what choices will users make? Currently, the growth of the mobile medical market ensures residents’ medical treatment is convenient and accessible, as well as injecting fresh vigor into their health levels. However, the mobile medical market lacks corresponding rules, regulations, and oversight mechanisms, resulting in market instability and a crisis of trust among residents, causing residents to refuse to use mobile medical services, and limiting the mobile medical market’s growth prospects and residents’ health levels. As a result, the development of the mobile medical market and residents’ rational behavior choices have a strong behavior game link. The goal is to investigate the mechanisms that influence the decision-making behavior of mobile medical businesses on user choice, which will not only help mobile medical businesses to make better decisions but will also encourage more users to choose and utilize mobile medical businesses. It will be fascinating and useful in literature by providing decision-making behavior of mobile medical businesses according to the user selection method.

The study summarizes and analyzes the existing literature on mobile medical development, business decision-making behavior, and user selection behavior, excavates the research problems and puts forward research hypotheses to build an evolutionary game model; and then solves the model and analyzes an example to obtain the decision-making behavior of mobile medical businesses based on user selection.

## Literature Review

The study on mobile medical businesses can be traced back to the two-way TV medical system. It was proposed by Wittson in the 1950s (Guo et al., 2012) and had gradually become the focus and hotspot of academic circles. International medical and health member organizations define mobile medicine as providing medical services and information using mobile phones, personal digital assistants (PDAs), medical sensors, wearable devices, etc., promoting the rational use of high-quality medical resources, enabling patients to find appropriate expert treatment and health consulting services in a convenient state, and improving the medical environment and medical experience (Britton et al., 2014).

At present, the research on the development of mobile medical businesses mainly discusses two aspects: application technology and business model. In terms of application technology, as a technology-dependent industry, mobile healthcare needs strong technical support in the integration and analysis of offline medical resources and user information security (Nian et al., 2013). China’s mobile medical technology is developing rapidly. Still, there are also some obstacles such as the lack of connectivity between various medical networks, the lack of product differences, and the lack of data security capabilities. In terms of business model, most mobile medical manufacturers worldwide provide personalized services and differentiated goods, such as drug dictionaries, diabetes management systems, and appointment services. Their revenue is mainly derived from pharmaceutical companies, insurance companies, hospitals, and other stakeholders (Heather et al., 2010). User preference and usage are important factors in the growth of the mobile medical market (Muro-Culebras et al., 2021). Furthermore, there is a significant positive association between the number of users who download and utilize mobile medical apps and the quality and evaluation outcomes of mobile medical products (Galetsi et al., 2021).

China has explored and innovated the business model of mobile medical businesses, and a series of business software apps, such as doctor Chunyu (consultation), Meiyou (health detection), good pharmacist (medical e-commerce), and micro medicine (online registration), have emerged. Since China’s pharmaceutical price system is not yet market-oriented, traditional medical institutions, such as the dominant body of resources, are almost reluctant to cut their own interests.

Research on the decision-making behavior of mobile medical businesses shows that due to the significant information asymmetry in the medical service market, medical service providers often have induced demand because of their own information advantages (Meyer, 2016). To obtain immediate profits, some app businesses seek attention by pushing eye-catching and novel advertisements to users and covering up their induced use by spreading health information and popularizing health knowledge. The intention of consumers to buy specific goods leads to the inconsistency in economic interests between the supplier and the demander, even conflicts, or disputes (Sajid et al., 2008). Regarding information security, mobile medical apps monitor users’ real-time activities, and businesses control users’ dynamic information. If the information is leaked, it will bring certain spiritual or property losses to users (Boruff and Storie, 2014). And owing to the lack of related policies and regulations system, the government’s supervision is insufficient, and the authority and professionalism of health information are not guaranteed. In the case of medical disputes, the responsibility relationship between users and app businesses is not clear; hence the decision-making behavior of mobile medical merchants will have a great impact on user selection behavior, which will restrict the development of the mobile medical market (Min et al., 2019).

The academic community’s choice behavior of mobile medical users mostly depends on the user’s personal factors, such as the user’s age (Fung et al., 2006), income (Fogel et al., 2002), educational background (Tian and Robinson, 2008), and preference (Wilson and Lankton, 2004), which have a certain impact on their willingness to choose the mobile medical business, and some scholars have also studied the cost (Mancini et al., 2006) and social impact (Xue et al., 2012) of mobile healthcare. Gefen et al. (2008) constructed a basic framework of user choice, including integrity, ability, and goodwill from the perspective of user trust in the network environment through empirical research. Akter et al. (2011) built a dimension model including structural factors, individual personality factors, and the reputation of mobile medical businesses. They analyzed the user’s choice behavior of mobile medical businesses from the trust. Some studies have also pointed out that consumers’ trust plays a significant intermediary role in user choice and sustainable development of mobile healthcare (Free and Phillips, 2013; Xie et al., 2017).

Generally speaking, most scholars have studied the development mode of mobile healthcare, business decision-making behavior, and the influencing factors of user choice, while there is little discussion on the influence of business decision-making behavior on the user choice behavior and the influence of business decision-making behavior and user choice behavior on the long-term development of mobile healthcare. Therefore, this study will build a decision-making behavior model of mobile medical businesses based on user selection, focusing on game theory to explore the decision-making behavior of mobile medical companies and the strategic mechanism of user selection. Based on this, we will further include the person’s role in the government bureau and systematically explore an organized regulatory mechanism of the government when the person in the bureau participates and meets certain conditions. On this basis, this paper deeply explores the impact mechanism of user selection behavior on the mobile medical market.

## Hypothesis and Models

### Methodology

The research aim in traditional classical game theory is often total rationality, although it is unusual to discover completely rational people in actual life. Most people or groups are with limited rationality, and limited rational people cannot find the best approach at any given point during the decision-making process. It is frequently a lifelong process of learning and evolution. Continuous self-adjustment, optimization, and improvement are also consistent with Darwin’s view of biological evolution. Darwin’s theory of biological evolution is the foundation of the evolutionary game theory. It is the outcome of rethinking classical game theory’s entire rational hypothesis. The evolutionary game theory assumes that people are rational, which overcomes the limitations of classical game theory’s complete rationality hypothesis, and believes that all game players, even those with limited rationality, will seek the final stability strategy through dynamic processes such as continuous learning, imitation, and experimentation.

As a result, the process of bounded rationality, inadequate information, and recurrent games is more by reality. Using evolutionary game theory to understand and explain real-world problems will provide more benefits. The evolutionary game theory has been consistently supplemented and refined in recent years, and it is now widely applied in practice, such as in the research of corporate social responsibility implementation mechanisms, e-government, and the eco industry, among other things. This study applies evolutionary game theory to the impact of user selection on the decision-making behavior of Chinese mobile medical firms, which can not only enhance evolutionary game theory but also develop and innovate its application.

### Hypothesis

From the above analysis, we can conclude that the rational economic man is the starting point for mobile medical businesses and users to take strategic measures, and the goal of promoting the sustainable development of mobile medical businesses is to build an excellent mobile medical environment and user trust mechanism, which rely on the macro-control measures of the government. Therefore, the following hypotheses are proposed:

(1) The essence of the public welfare of medical institutions requires mobile medical businesses to create a good medical environment for users. In addition, the characteristics of mobile medical commercialization and information asymmetry between doctors and patients will lead to user information leakage and induced demand, which will easily lead to a bad mobile medical environment. Therefore, it is assumed that the parameter *r*_*1*_ was the revenue per unit of service provided by mobile medical businesses; the parameter *c*_*1*_ was the action cost of creating a good environment for mobile medical businesses; the parameter *c*_*2*_ was the cost of creating adverse environments such as induced demand and information leakage for mobile medical businesses (*c*_1_ > *c*_2_); the parameter *p*_*1*_ was the probability of creating an adverse environment for mobile medical businesses, and the parameter *q* was the number of mobile medical services.

(2) As an individual’s decision-making is always accompanied by the process of information screening and focusing, after information filtering, the individual’s perceived risk becomes larger, and behavior will be more cautious (Free and Phillips, 2013). Suppose that the parameter *c*_*3*_ was the money and information cost for users to choose to download, register, and apply each unit of mobile medical APP, *x* was the proportion of filtering information for users. The parameter *c*_*4*_ was the additional cost that users pay for filtering information. We set the probability that users will be affected by the bad environment of businesses after filtering information as 0. In contrast, users will be affected by the bad environment if they do not filter information, and the revenue of each unit of mobile medical service was *r*_*2*_, including the value of time, money, convenience, and so on. The parameter *c*_*5*_ was the cost of purchasing unnecessary goods or information leakage caused by the adverse environment after the user downloads the mobile medical application. At this time, the users have two choices: one is to save unnecessary trouble and choose to let it go and the other is to take negotiation measures or legal means to protect their rights and interests. The parameter *p*_*2*_ was the probability that users choose to protect their rights because of the adverse environment created by mobile medical businesses. The parameter *r*_*3*_ was the compensation given by the merchants after the adverse environment of mobile medical protects the users.

(3) The development of the mobile medical market is highly valued by the government. Therefore, it can be assumed that the government gives certain incentive measures to mobile medical businesses, including financial subsidies and relevant policies. It is believed that these incentive measures can be converted into the monetary value of businesses *h*_1_.

### Models

#### Non-government Intervention Model

Non-government intervention means that the government does not participate in the supervision of mobile health businesses, and the market mechanism mainly dominates its operation mode. The following formula can express the expected revenue of mobile medical businesses.


$$E_{m}^{0}=\left(1-p_{1}\right)\,\left(r_{1}\,q+h_{1}-c_{1}\right)+p_{1}\,\left[\left(1-p_{2}\right)\,\left(r_{1}\,q+h_{1}-c_{2}\right)+p_{2}\,\left(r_{1}\,q+h_{1}-c_{2}-r_{3}\right)\right]$$

The expected revenue of users can be expressed as the expected revenue.


$$\begin{array}{c} E_{u}^{0}=\left(1-p_{1}\right)\,x\,\left(1-p_{2}\right)\,\left(r_{2}\,{\text{q–c}}_{3}-c_{4}\right)+\left(1-p_{1}\right)\,\left(1-x\right)\,\left(1-p_{2}\right)\\ \left(r_{2}{\text{q–c}}_{3}\right)+p_{1}\left(1-x\right)[p_{2}\left(r_{2}{\text{q–c}}_{3}-c_{5}+r_{3}\right)+\left(1-p_{2}\right)(r_{2}\\ +{\text{q–c}}_{3}-c_{5})]p{}_{1}x(1-p_{2})(r_{2}{\text{q–c}}_{3}-c_{4}) \end{array}$$

#### Policy Supervision Model

The mobile medical industry is a professional and high threshold industry, and it needs to be strictly controlled. However, China’s mobile medical policies and regulations are not perfect, and in some aspects are still blank, and the field of mobile medical businesses involves many complicated interests. Therefore, to ensure a good operating environment for mobile medical businesses, it is assumed that the government will conduct random inspections on mobile medical businesses from time to time, and the probability of random inspection was *k*. For the convenience of analysis, it is assumed that once the random inspection is carried out, it is sure to find out whether the businesses have created a bad environment, and the cost of the random inspection was *c*_*g*_. If the app creates a bad environment, it will be ordered to rectify and give corresponding punishment to the business, the amount of punishment was *m*.

Suppose that after the government supervision, the overall environment of the mobile medical market is improved, the cost of users’ filtering information is reduced to (1), and the compensation after rights protection is increased to (2). Then the expected revenue of mobile medical businesses under government intervention can be expressed as follows:


$$\begin{array}{c} E_{m}=\left(1-p_{1}\right)\left(r_{1}q+h_{1}-c_{1}\right)+p_{1}\left(1-k\right)[\left(1-p_{2}\right)\\ \left(r_{1}q+h_{1}-c_{2}\right)+p_{2}\left(r_{1}q+h_{1}-c_{2}-r_{3}\right)]+p{}_{1}k[\left(1-p_{2}\right)\\ \left(r_{1}q+h_{1}-c_{2}\right)+p_{2}\left(r_{1}q+h_{1}-c_{2}-r_{4}\right)-m] \end{array}$$

The expected revenue of users can be expressed as follows:


$$\begin{array}{c} E_{u}=\left(1-p_{1}\right)\,x\,\left(1-p_{2}\right)\,\left(r_{2}\,{\text{q–c}}_{3}-c_{6}\right)+\left(1-p_{1}\right)\,\left(1-x\right)\,\left(1-p_{2}\right)\\ \left(r_{2}{\text{q–c}}_{3}\right)+p_{1}\left(1-x\right)[p_{2}\left(r_{2}q-c_{3}-c_{5}+r_{4}\right)+\left(1-p_{2}\right)\\ \left(r_{2}{\text{q–c}}_{3}-c_{5}\right)]+p{}_{1}p{}_{2}x(r_{2}{\text{q–c}}_{3}-c_{6}+r_{4})+p{}_{1}(1-p_{2})x\\ \left(r_{2}\,{\text{q–c}}_{3}-c_{6}\right) \end{array}$$

The income function of government can be expressed as follows:


$$E_{g}=\left(1-p_{1}\right)\,k\,\left(-c_{g}\right)+p_{1}\,k\,\left(m-c_{g}\right)$$

### Results

#### Non-government Intervention

In the absence of government intervention, mobile medical businesses usually decide whether to create a good environment according to the rights of users.


$$\{{}_{p_{2}^{{}^{*}}=\frac{c_{1}-c_{2}}{r_{3}}}^{p_{1}^{{}^{*}}=\frac{r_{2}\,{\text{q–c}}_{3}-x\,c_{4}}{r_{2}\,{\text{q–c}}_{3}+r_{3}-x\,\left(r_{3}+r_{2}\,{\text{q–c}}_{3}\right)}}$$

It can be seen from the above formula that for the sake of its own operational development and the pursuit of its own interests, mobile medical businesses will create a bad mobile medical environment with the probability of $p_{1}^{{}^{*}}$, while users will choose to protect their rights with the probability of $p_{2}^{{}^{*}}$. Substituting $p_{1}^{{}^{*}}$ and $p_{2}^{{}^{*}}$ into the income function expression, we can find the utility of mobile medical businesses $U_{1}\,\left[E_{m}^{0}\,\left(p_{1}^{{}^{*}},p_{2}^{{}^{*}}\right)\right]$ and users $U_{2}\,\left[E_{u}^{0}\,\left(p_{1}^{{}^{*}},p_{2}^{{}^{*}}\right)\right]$ as follows:


$$U_{1}\,\left[E_{m}^{0}\,\left(p_{1}^{{}^{*}},p_{2}^{{}^{*}}\right)\right]=r_{1}\,q+h_{1}-c_{1}$$

$$\begin{array}{c} U_{2}\,\left[E_{u}^{0}\,\left(p_{1}^{{}^{*}},p_{2}^{{}^{*}}\right)\right]=\left(1-p_{1}^{{}^{*}}\right)\,\left(1-p_{2}^{{}^{*}}\right)\,\left(-x\,c_{4}+r_{2}\,{\text{q–c}}_{3}\right)+p_{1}^{{}^{*}}\,\left(1-x\right)\\ \left(p_{2}^{{}^{*}}\,r_{3}+r_{2}\,{\text{q–c}}_{3}-c_{5}\right)+p_{1}^{{}^{*}}\,x\,\left(1-p_{2}^{{}^{*}}\right)\,\left(r_{2}\,{\text{q–c}}_{3}-c_{4}\right) \end{array}$$

#### Government Regulation

When the government participates in the supervision, the government’s supervision is non-utilitarian. In the supervision process, it will consider the cost problem. Still, it does not take the maximization of income as the ultimate goal, so its income function does not constitute a constraint. Therefore, the reverse induction method can be used to solve the problem, and the probability of the mobile medical business to create a bad environment and the users’ rights protection is, respectively, as follows:


$$\{{}_{p_{2}^{**}=\frac{{}_{c_{1}-c_{2}-k\,m}}{\left(1-k\right)\,r_{3}+k\,r_{4}}}^{p_{1}^{**}=\frac{r_{2}\,{\text{q–c}}_{3}-x\,c_{6}}{{}_{r_{2}\,{\text{q–c}}_{3}-x\,c_{6}+r_{4}}}}$$

By substituting the probabilities $p_{1}^{**}$ and $p_{2}^{**}$ of the above parties to maximize personal income into the income function, we can obtain the utility functions $U_{3}\,\left[E_{m}\,\left(p_{1}^{**},p_{2}^{**}\right)\right]$ and $U_{4}\,\left[E_{u}\,\left(p_{1}^{**},p_{2}^{**}\right)\right]$ of mobile medical businesses and users using the following models.


$$U_{3}\,\left[E_{m}\,\left(p_{1}^{**},p_{2}^{**}\right)\right]=r_{1}\,q+h_{1}-c_{1}+p_{1}^{**}\,\left(c_{1}-c_{2}-p_{2}^{**}\,r_{3}\right)+p_{1}^{**}\,k\,\left(p_{2}^{**}\,r_{3}-p_{2}^{**}\,r_{4}+m\right)$$

$$\begin{array}{c} U_{4}\,\left[E_{u}\,\left(p_{1}^{**},p_{2}^{**}\right)\right]=\left(1-p_{{}^{1}}^{**}\right)\,\left(1-p_{2}^{**}\right)\,\left(-x\,c_{6}+r_{2}\,{\text{q–c}}_{3}\right)\\ +p_{1}^{**}\,\left(p_{2}^{**}\,r_{4}+r_{2}\,{\text{q–c}}_{3}-c_{5}\right)+p_{1}^{**}\,x\,\left(c_{5}-c_{6}\right) \end{array}$$

#### Comparison of Results

After comparing and analyzing the probability of creating a good environment, the probability of users’ rights protection, and the utility changes of each participant under the two models, we can find the following equation:


$$p_{1}^{**}-p_{1}^{*}=\frac{r_{2}\,{\text{q–c}}_{3}-x\,c_{6}}{\left(r_{2}\,{\text{q–c}}_{3}-x\,c_{6}+r_{4}\right)}-\frac{r_{2}\,{\text{q–c}}_{3}-x\,c_{4}}{r_{2}\,{\text{q–c}}_{3}+r_{3}-x\,\left(r_{3}+r_{2}\,{\text{q–c}}_{3}\right)}<0\,p_{2}^{**}-p_{2}^{*}=\frac{c_{1}-c_{2}-k\,m}{\left(1-k\right)\,r_{3}+k\,r_{4}}-\frac{c_{1}-c_{2}}{r_{3}}<0$$

$$U_{3}-U_{1}=p_{1}^{**}\,\left(c_{1}-c_{2}-p_{2}^{**}\,r_{3}\right)+p_{1}^{**}\,k\,\left(p_{2}^{**}\,r_{3}-p_{2}^{**}\,r_{4}+m\right)>0$$

$$\begin{array}{c} U_{4}-U_{2}=\left(1-p_{1}^{**}\right)\,\left(1-p_{2}^{**}\right)\,\left(-x\,c_{6}+r_{2}\,{\text{q–c}}_{3}\right)+p_{1}^{**}\\ \left(p_{2}^{**}\,r_{4}+r_{2}\,{\text{q–c}}_{3}-c_{5}\right)+p_{1}^{**}\,x\,\left(c_{5}-c_{6}\right)-\left(1-p_{1}^{*}\right)\\ \left(\left(1-p_{2}^{*}\right)-x\,c_{4}+r_{2}\,{\text{q–c}}_{3}\right)+p_{1}^{*}\,\left(1-x\right)\\ \left(p_{2}^{*}\,r_{3}+r_{2}\,{\text{q–c}}_{3}-c_{5}\right)+p_{1}^{*}\,x\,\left(1-p_{2}^{*}\right)\,\left(r_{2}\,{\text{q–c}}_{3}-c_{4}\right) \end{array}$$

From this, we can get Proposition 1.

Proposition 1: When the government participates in the supervision of the mobile medical environment, the probability of users choosing to protect their rights decreases, while the probability of mobile medical businesses creating a bad medical environment decreases, the expected utility increases.

By analyzing the sign *U*_4_−*U*_2_, we can see that when *U*_4_−*U*_2_ > 0, the government’s supervision of mobile medical environment can improve the average utility level of users; on the contrary, when *U*_4_−*U*_2_ < 0, it will reduce the utility of users choosing mobile medical.

If other parameters remain unchanged, the relationship among $p_{1}^{*}$, $p_{1}^{**}$, and *x* was analyzed.


$$\frac{\partial p_{1}^{*}}{\partial x}=\frac{\begin{array}{c} -c\,4\,\left[r_{2}\,{\text{q–c}}_{3}+r_{3}-x\,\left(r_{3}+r_{2}\,{\text{q–c}}_{3}\right)\right]\;\\ +\left(r_{2}\,{\text{q–c}}_{3}-x\,c_{4}\right)\,\left(r_{3}+r_{2}\,{\text{q–c}}_{3}\right)\; \end{array}}{{\left[r_{2}\,{\text{q–c}}_{3}+r_{3}-x\,\left(r_{3}+r_{2}\,{\text{q–c}}_{3}\right)\right]}^{2}}$$

$$\frac{\partial p_{1}^{**}}{\partial x}=\frac{-c_{6}\,r_{4}}{{\left(r_{2}\,{\text{q–c}}_{3}-x\,c_{6}+r_{4}\right)}^{2}}$$

From this, we can get Proposition 2.

Proposition 2: In the absence of government intervention, the increased probability of information filtering after users choose to apply mobile medical businesses will increase the probability of creating a bad environment for mobile medical businesses. Under the condition of government supervision, when the probability of users’ filtering information increases, the probability of medical mobile companies creating a bad environment decreases.

If other parameters remain unchanged, the relationship among $p_{1}^{**}$, $p_{2}^{**}$, and *k* is analyzed.


$$\frac{\partial p_{1}^{**}}{\partial k}=0$$

$$\frac{\partial p_{2}^{**}}{\partial k}=\frac{-m\,r_{3}+\left(c_{1}-c_{2}\right)\,\left(r_{3}-r_{4}\right)}{{\left[\left(1-k\right)\,r_{3}+r_{4}\,k\right]}^{2}}$$

From this, we can get Proposition 3.

Proposition 3: After the government participates in the supervision of mobile medical environments, the probability of random inspection of mobile medical businesses increases, which has no impact on the probability of whether the businesses create a good environment, but will reduce the probability of users choosing to protect their rights.

## Analysis

The probability of creating a good environment for mobile medical businesses and the likelihood of users choosing to protect their rights after being affected by the bad environment of mobile medical businesses have been obtained through strict analysis and derivation. The probability changes with the probability of users’ filtering information and the probability of government sampling mobile medical environment. As the expression of the utility function is complex, it is difficult to make a direct comparative analysis. Therefore, the study mainly focuses on the impact of user’s awareness of filtering information, the sampling degree of the government to mobile medical businesses, and the punishment of the government to create a bad environment for mobile medical businesses on creating a good environment for mobile medical businesses through numerical simulation.

According to the survey of practical applications and the download of some mobile apps in China, the average income of each unit of service provided by mobile medical businesses is 10 Yuan, the unit action cost of creating a good environment for mobile medical businesses is 5 Yuan, and the unit action cost of creating a bad environment is 3 Yuan. Suppose that the cost *c*_*3*_ for users to register, download, and apply each unit of the mobile medical app is 12 Yuan, the cost *c*_*4*_ for filtering information is 2 Yuan, and the income *r*_*2*_ is 15 Yuan. When users are affected by an adverse environment, the cost *c*_*5*_ is 5 Yuan (at this time, the income does not change), and the business compensation *r*_*3*_ is 3 Yuan. The unit fund *h*_*1*_ invested by the government to encourage the development of mobile healthcare is 1 Yuan. For the convenience of calculation, we assume that *q* is 1, that is, the behavior choices and revenue changes of mobile medical businesses and users after users apply each unit of medical services.

**FIGURE 1:**
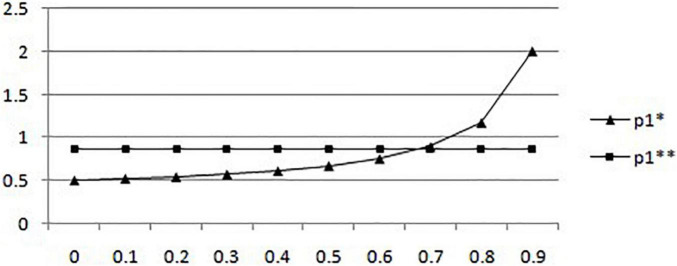
In the situation of absence of government intervention and government regulation, the probability of mobile medical businesses creating a good environment changes with the probability of users filtering information.

(1) Considering the situation of non-government intervention and government supervision, the probability of mobile medical businesses creating a bad environment changes with the probability of users’ filtering information. The study further assumes that the cost *c*_*6*_ of users’ filtering information after government supervision is 1 Yuan, and the compensation *r*_*4*_ after rights protection is 4 Yuan. The probability of the government sampling mobile medical businesses is 1, and the probability of users’ filtering information increases from 0.1 to 0.99, and the results are shown in Figure 1.

According to Figure 1, in the case of non-government intervention, the probability of mobile medical businesses creating a bad environment increases with a surge in the likelihood of users’ filtering information. When the proportion of users’ filtering information is greater than 0.7, the probability of mobile medical businesses creating a good environment tends to be 1. It means that in a given situation, an upsurge of the possibility of patients choosing to filter mobile medical information will affect the economic profits of the business, so the probability of mobile medical business to create a bad environment will be significantly improved with the increase in the probability of users’ filtering information. On the contrary, in the case of government participation in mobile medical supervision, when the probability of users filtering mobile medical information increases, the probability of mobile medical businesses creating a bad medical environment decreases. However, the numerical results obtained by the example (which example are you referring to) show that the downward trend is not obvious, which shows that when the government participates in the supervision of the mobile medical environment, the effect of users choosing to filter information on reducing the adverse environment created by mobile medical businesses is not significant.

**FIGURE 2:**
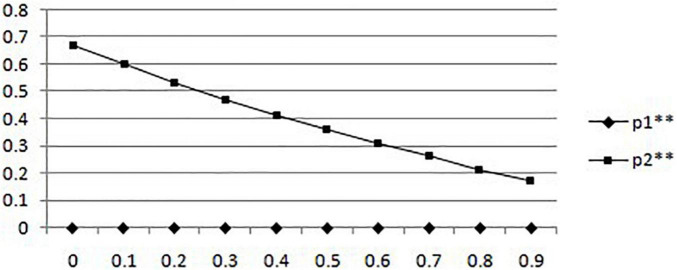
In the situation of government supervision, the probability of creating a good environment for mobile medical businesses and the probability of users rights protection change with the probability of government sampling.

(2) In the case of government supervision, the probability of mobile medical businesses creating a bad environment and the probability of users choosing to protect their rights change with the probability of government sampling mobile medical services. Suppose that the government’s unit penalty for unqualified mobile medical businesses after sampling is 1.5 Yuan, while the probability of users choosing to filter mobile medical information is 0 and the probability of users choosing to filter mobile medical information is 1, the probability of government sampling increases from 0 to 0.99, and the results are shown in Figure 2.

It can be seen that when the government participates in the supervision of mobile healthcare, the change in sampling probability of mobile healthcare business cannot effectively improve the probability of business creating a bad environment. While the probability of the government’s sampling of mobile medical businesses increases, the probability of users choosing to protect their rights after being affected by the adverse mobile medical environment increases. It shows that under the assumption of a given situation after the government participates in mobile medical supervision, the sampling behavior of the government cannot effectively affect the behavior choice of mobile medical businesses. On the contrary, it can effectively enhance the awareness of the rights protection of users.

**FIGURE 3:**
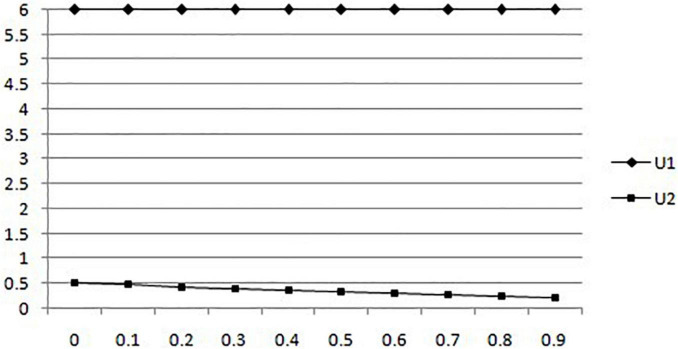
In the absence of government intervention, the expected utility of mobile medical businesses and users changes with users’ choice of filtering information.

(3) Considering the changes in the expected utility of mobile medical businesses and users with the probability of users choosing to filter information without government intervention, the probability of users choosing to filter information increases from 0 to 0.99, and the result is shown in Figure 3.

According to Figure 3, we can find that the increase in the probability of information filtering will not affect the expected utility of mobile medical businesses when users choose to apply mobile medical businesses. It is consistent with the previous results that the probability of merchants creating a bad environment increases after information filtering. In addition, while the probability of information filtering increases, the expected utility of users will be reduced.

**FIGURE 4:**
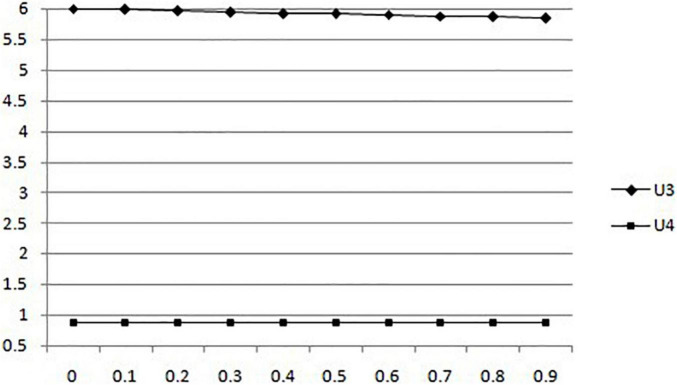
In the situation of government supervision, the changes of expected utility of mobile medical businesses and users with the probability of government sampling.

(4) Considering the changes in the expected utility of mobile medical businesses and users with the probability of government sampling under government supervision, the probability of government sampling increased from 0 to 0.99, and the results are shown in Figure 4.

According to Figure 4, we can find that the government’s participation in mobile medical supervision will reduce the utility of mobile medical enterprises. But when the probability of government sampling increases, the expected utility of users is not affected.

The above analysis tests the basic assumptions and model solution results according to real cases, and the two research results are consistent, indicating the reliability of the research results.

## Discussion

First, the establishment and improvement of relevant laws and regulations is the basis to ensure the orderly operation of the mobile medical market. It not only helps to enhance the expected utility of users and businesses in the application of mobile medical businesses and business development processes but also helps to enhance the overall social benefits. It also plays an essential role in alleviating the problem of “expensive and difficult to see a doctor” and “costly and expensive in seeing a doctor” existing in the current situation of China’s medical service system.

In addition, government regulation is not only an effective measure to maintain a good mobile medical environment but also an important way to improve user efficiency and the expected utility of businesses. It is also the fundamental guarantee to promote the healthy, stable, and sustainable development of the mobile medical industry.

Moreover, good publicity and education can protect the fundamental interests of users and businesses. To improve the medical environment of residents as the core, the health level of residents must be improved as the goal, so that users and mobile medical businesses can make clear their behavior choice strategies under reasonable conditions.

The main contribution of this study was to analyze the game relationship among users’ selection behavior, mobile medical business decision-making behavior, and government supervision behavior in the process of mobile medical development through the evolutionary game method. The research conclusion provides theoretical support for promoting the growth of the mobile medical market, improving the quality of mobile medical service, and ensuring the accessibility of user health. At the same time, it also provides practical suggestions for strengthening government functions and standardizing business behavior in the process of mobile medical development.

## Conclusion

In the case of non-government intervention, users will gain an excellent mobile medical environment in a short time by filtering and screening mobile medical information for their own development and the pursuit of economic interests, and mobile medical businesses will further create a bad mobile medical environment. In the long run, in the situation of non-government intervention, users’ filtering information will not affect the expected utility of businesses but will damage users’ own expected utility to a certain extent. Users’ filtering information will not harm the expected utility of companies in the case of non-government interference but will damage the expected utility of users to some degree in the long term.

In government supervision, the mobile medical environment is effectively improved, the probability of users’ rights protection is reduced, and the expected utility of mobile medical businesses is improved. When the probability of users’ filtering information increases, the probability of medical mobile companies creating a bad environment decreases slightly, but the downward trend is not apparent. When the probability of government monitoring the mobile medical environment increases, the expected utility of mobile medical businesses decreases. In contrast, the expected utility of users is not affected, and the probability of users’ choosing to protect their rights increases after being affected by the adverse mobile medical environment, but it has no effect on the probability of mobile medical businesses creating an adverse environment. This shows that government regulation can effectively control the mobile medical environment. Once users are affected by the adverse environment, they will protect their rights to seek more economic compensation.

## Data Availability Statement

The original contributions presented in the study are included in the article/supplementary material, further inquiries can be directed to the corresponding author/s.

## Author Contributions

XX conceived the idea, collected the data, and revised the manuscript in line with the objectives. JW analyzed the data and drafted the manuscript. LZ sequentially aligned the parts of the research manuscript. HA collected data and analyzed the data. All authors read and approved the final manuscript.

## Conflict of Interest

The authors declare that the research was conducted in the absence of any commercial or financial relationships that could be construed as a potential conflict of interest.

## Publisher’s Note

All claims expressed in this article are solely those of the authors and do not necessarily represent those of their affiliated organizations, or those of the publisher, the editors and the reviewers. Any product that may be evaluated in this article, or claim that may be made by its manufacturer, is not guaranteed or endorsed by the publisher.
